# 2249: Gene expression signatures of acute respiratory syncytial virus infection in pediatric patients reveals insight into clinical pathogenesis

**DOI:** 10.1017/cts.2017.213

**Published:** 2018-05-10

**Authors:** Darrell Dinwiddie, Walter Dehority, Kurt C. Schwalm, Raymond J. Langley, Stephen A. Young, Joshua L. Kennedy

## Abstract

OBJECTIVES/SPECIFIC AIMS: Respiratory viruses cause enormous medical burden, yet many of the specific virus and host genetic factors that impact pathogenesis are still largely unknown or poorly understood. To better understand the drivers of both acute clinical pathogenesis and long-term impacts, such as the development of asthma, we investigated the host response to respiratory syncytial virus (RSV) infections in pediatric patients. METHODS/STUDY POPULATION: We collected nasopharyngeal swabs from 32 pediatric patients with acute RSV infection. The swabs represented a mixed cell population including epithelial and immune cells at the active site of infection. Unbiased RNA sequencing with ribosomal RNA depletion allowed the simultaneous detection of host gene expression and RSV infection. We sequenced samples 2×75 bp on an Illumina NextSeq 500. Sequences were mapped to the human genome using the TopHat 2 aligner and FPKM estimation of reference genes and transcripts and assembly of novel transcripts were conducted with Cufflinks 2. RESULTS/ANTICIPATED RESULTS: During acute RSV infection we identified 7343 genes that were significantly expressed. Pathway analysis using KEGG revealed significant upregulation of pathways involved in innate immune response infection, ribosome function, oxidative phosphorylation, spliceosome and autoimmune disorders. We found high levels of innate immune response genes including CXCL8, IFITM1, IFITM2, IFITM3, IL1RN, and ISG15. In comparing RSV subtype A to RSV B we found significant differential expression of multiple noncoding RNAs. DISCUSSION/SIGNIFICANCE OF IMPACT: Examination of the host gene response during acute RSV infections, yielded important insight into the mechanisms that cause clinical pathogenesis and may provide understanding of the mechanisms that lead to known long-term impacts, such as the development of asthma. Together, this data may be used to guide clinical treatment and management decisions for children with severe RSV infections.

